# ARE RURAL OLDER ADULTS READY TO ADOPT AUTONOMOUS VEHICLES (AV)? PREDICTORS OF ATTITUDES TOWARD AV IN RURAL AREAS

**DOI:** 10.1093/geroni/igad104.3075

**Published:** 2023-12-21

**Authors:** Mojgan Zoaktafi, Tyler Gandy, Jong Sung Yoon

**Affiliations:** University of South Dakota, Vermillion, South Dakota, United States; University of South Dakota, Vermillion, South Dakota, United States; University of South Dakota, Vermillion, South Dakota, United States

## Abstract

Transportation for older adults residing in rural areas is particularly vital for maintaining their everyday activities including access to health services and community activities. Given unique features of rural areas (e.g., limited public transportation, greater distances to travel but less traffic), autonomous vehicles (AV) can be an innovative solution to rural older adults. Nevertheless, attitudes toward AV can be a significant barrier for older adults to adopt it. In fact, older adults in rural areas tend to hold different attitudes toward and experiences with new technologies. The current study explored how individual difference variables (i.e., age group, gender, technology proficiency, & personality traits) predict attitudes toward AV in rural areas. We first conducted a factor analysis on a 16-item attitude survey (collected from rural areas in USA) which uncovered three factors: concern with AV, willingness to adopt AV, confidence on human driving over AV. ANOVAs on the attitudinal factors showed that older adults (60+ yrs, n = 45) had a lower willingness to adopt AV than young adults (17-21 yrs, n = 47) despite a lower confidence on human driving over AV. There was no age group difference in concern with AV. Multiple regressions supported that technology proficiency can be another significant predictor of willingness to adopt AV over and above other individual difference factors (i.e., age groups, gender, & personality). These results indicate that older adults in rural areas have complex and ambivalent attitudes toward AV, which suggest different approaches to the dissemination of AV technology for them.

